# Applying the behavioural change wheel to guide the implementation of a biopsychosocial approach to musculoskeletal pain care

**DOI:** 10.3389/fpain.2023.1169178

**Published:** 2023-05-09

**Authors:** Wendy Ng, Darren Beales, Daniel F. Gucciardi, Helen Slater

**Affiliations:** ^1^ Curtin School of Allied Health, Curtin University, Perth, WA, Australia; ^2^ Curtin enAble Institute, Curtin University, Perth, WA, Australia

**Keywords:** biopsychosocial, behaviour change techniques, behaviour change techniques taxonomy version 1, capability opportunity motivation-Behaviour, healthcare professionals, musculoskeletal pain, theoretical domains framework

## Abstract

Achieving high value, biopsychosocial pain care can be complex, involving multiple stakeholders working synergistically to support the implementation of quality care. In order to empower healthcare professionals to assess, identify and analyse biopsychosocial factors contributing to musculoskeletal pain, and describe what changes are needed in the whole-of-system to navigate this complexity, we aimed to: (1) map established barriers and enablers influencing healthcare professionals' adoption of a biopsychosocial approach to musculoskeletal pain against behaviour change frameworks; and (2) identify behaviour change techniques to facilitate and support the adoption and improve pain education. A five-step process informed by the Behaviour Change Wheel (BCW) was undertaken: (i) from a recently published qualitative evidence synthesis, barriers and enablers were mapped onto the Capability Opportunity Motivation-Behaviour (COM-B) model and Theoretical Domains Framework (TDF) using “best fit” framework synthesis; (ii) relevant stakeholder groups involved in the whole-of-health were identified as audiences for potential interventions; (iii) possible intervention functions were considered based on the Affordability, Practicability, Effectiveness and Cost-effectiveness, Acceptability, Side-effects/safety, Equity criteria; (iv) a conceptual model was synthesised to understand the behavioural determinants underpinning biopsychosocial pain care; (v) behaviour change techniques (BCTs) to improve adoption were identified. Barriers and enablers mapped onto 5/6 components of the COM-B model and 12/15 domains on the TDF. Multi-stakeholder groups including healthcare professionals, educators, workplace managers, guideline developers and policymakers were identified as target audiences for behavioural interventions, specifically education, training, environmental restructuring, modelling and enablement. A framework was derived with six BCTs identified from the Behaviour Change Technique Taxonomy (version 1). Adoption of a biopsychosocial approach to musculoskeletal pain involves a complex set of behavioural determinants, relevant across multiple audiences, reflecting the importance of a whole-of-system approach to musculoskeletal health. We proposed a worked example on how to operationalise the framework and apply the BCTs. Evidence-informed strategies are recommended to empower healthcare professionals to assess, identify and analyse biopsychosocial factors, as well as targeted interventions relevant to various stakeholders. These strategies can help to strengthen a whole-of-system adoption of a biopsychosocial approach to pain care.

## Introduction

Engel's biopsychosocial model (1), has provided a blueprint for contemporary care of chronic pain disorders (2–10). However, there are significant challenges putting this model into clinical practice (11, 12). Pain is complex with multidimensional (biological, psychological and social) factors interacting to influence the lived experience (3, 13), often with multimorbidity (chronic lifestyle illnesses and mental health illnesses) (14). This complexity makes comprehending and caring for each individual's needs as a whole person challenging for healthcare professionals.

Against this background, we previously systematically reviewed evidence and generated insights on the barriers and enablers to the adoption of the biopsychosocial model in musculoskeletal pain, spanning the whole-of-health. Our recent qualitative review included 25 studies and the perspectives of 413 healthcare professionals (15). There are multiple factors influencing healthcare professionals' adoption of the biopsychosocial model. At the micro-level (clinical interface), healthcare professionals' knowledge and skills, personal factors, their misconceptions of clinical practice guidelines, perception of patients' factors, and time can influence adoption of a biopsychosocial approach. At the meso-level (health service provision), clinical practice guideline formulation, the availability and alignment of the clinical community, funding models, health service provision, resourcing, and workforce training issues may or may not adequately support the care. At the macro-level (health system), health policy, organizational, and social factors can significantly affect and shape how care for musculoskeletal disorders is delivered. Further evidence for challenges to adoption come from another review that included 12 qualitative studies and the views of 113 physiotherapists showing that despite the positive changes with education, physiotherapists lack confidence to implement biopsychosocial pain care (16). These findings are supported by the modest effect of educational meetings on changing clinical practice behaviours and clinical outcomes (17–21). Re-design of educational efforts to address the micro-level barriers might facilitate healthcare professionals in adopting the model in pain care, while also leveraging the meso- and macro-level enablers.

Using behavioural science frameworks to understand human behaviour may provide insights into *how* to drive translation efforts to support effective design of behavioural interventions that target relevant audiences involved in pain care (22). Inferring from Engel's original frame of reference of the Biopsychosocial Model (1), our specification of the target behaviour is: Healthcare professionals (who) assessing, identifying and analysing biopsychosocial factors contributing to musculoskeletal pain (what), using authentic communication upon patient interview within a strong therapeutic alliance and critical clinical reasoning (how, with whom), during consultation in clinical practice (when, where). We are also interested in what the critical stakeholder groups within healthcare services and systems (meso- and macro-level) can do, to assist healthcare professionals to achieve specified target behaviours.

The Behaviour Change Wheel (BCW) is derived from 19 frameworks of behaviour change, and is a systematic process used for designing behavioural interventions (23, 24). Broadly, the process covers understanding the behaviour, identifying intervention options, and identifying content and implementation options (23, 24). At the hub of the wheel is the Capability Opportunity Motivation-Behaviour (COM- B) model, surrounded by nine intervention functions and seven policy categories (24). The Theoretical Domains Framework (TDF) expands on the COM-B components and provides a more detailed understanding of the cognitive, affective, social and environmental influences on behaviour (25). The COM-B and TDF can be used to understand behaviour at the individual, community and organizational levels (23), i.e., allows us to analyse necessary conditions internal to individuals, and the social and physical environment to achieve a specified target behaviour (24). This is well-aligned to investigating what can empower healthcare professionals to assess, identify and analyse biopsychosocial factors at the clinical-level, what can support them at the health service and policy levels (across multi-levels) (26). It is also worth noting the COM-B model and TDF have been used in the implementation of evidence-based recommendations of musculoskeletal conditions (27–30). The hypothesized relationship between the COM-B model components and intervention functions in the BCW allows a precise analysis of how to make the selection of interventions and policies (24), after which can then be linked to specific behaviour change techniques (BCTs) (24, 31). The BCW offered a comprehensive and solid theoretical foundation for the synthesis.

Thus, the aims of this study are:

(i) to map established barriers and enablers influencing healthcare professionals' adoption of a biopsychosocial approach to musculoskeletal pain (15) using theoretical frameworks of behaviour change (23–25, 27, 31), and in the process, identify the behavioural determinants that can support the adoption,
(ii) formulate a novel conceptual model (using concepts from the COM-B model and TDF) to outline the behavioural determinants, as an overview to a whole-of-health perspective to healthcare professionals' adoption of the biopsychosocial model, and
(iii) derive a framework of BCTs that characterise how various stakeholder groups can help improve current pain education training efforts to support healthcare professionals' adoption of biopsychosocial musculoskeletal pain care.

## Methods

**Figure 1:**
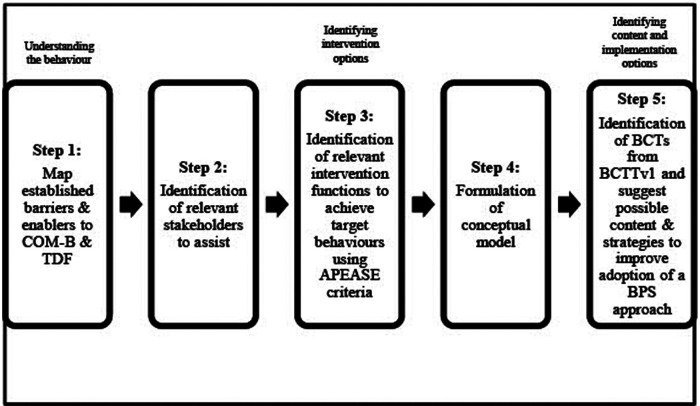
An Overview of the five-step process, informed by the behavioural change wheel (BCW). APEASE, affordability, practicability, effectiveness/cost-effectiveness, acceptability, side-effects/safety, equity; BCTs, behaviour change techniques; BCTTv1, behaviour change techniques taxonomy version 1; BPS, biopsychosocial; COM-B, capability opportunity motivation-behaviour model; TDF, theoretical domains framework.

We adopted a five-step process informed by the BCW (23, 24) (Figure 1) to synthesize the 46 subthemes and 14 main themes derived from our systematic review of the barriers and enablers influencing healthcare professionals' adoption of a biopsychosocial approach to musculoskeletal pain (15). These subthemes and main themes were therefore our data set used to apply the behavioural analysis. In each step, when discussion was necessary, iterative consensus was used to reach agreement within the research team (32). The characteristics and reflexivity of the research team members are described in Table 1. The team adopted our epistemological position as constructivist (33). Overall, the team has expertise that cuts across the micro-, meso- and macro-levels of healthcare, and proficiency with the biopsychosocial model, musculoskeletal pain, and the BCW process. Please note that all definitions and detailed description of terminologies related to the BCW are provided in the Supplementary Tables S1–S4.

**Table 1:** Characteristics & reflexivity of the research team.

Member	Background	Relevant experience	Years of research experience	Years of clinical experience	Years of teaching experience
WN	PhD candidate Clinician	Lead author of the qualitative systematic review preceding this study.	5^a^	15	–
		Musculoskeletal physiotherapist, breadth of experience spans adult and paediatric musculoskeletal pain/disorders, with clinical focus on chronic and complex musculoskeletal pain using a biopsychosocial approach.			
		Person with a lived experience of pain.			
DB	Clinician-Researcher	Practicing Specialist Musculoskeletal Physiotherapist (as awarded by the Australian College of Physiotherapists). Extensive clinical work including working in multidisciplinary teams to manage complex pain conditions and implementation of programs to enhance biopsychosocial patient care. Senior Research Fellow with broad research activities covering mechanistic understanding of clinical pain through to efforts to enhance the management of persistent pain and implementation of knowledge into practice.	22	30	22
		Educational experience with focus on the implantation of person-centered care that is biopsychosocial in nature, at the undergraduate and post-graduate levels.			
		President of the Australian Physiotherapy Association for the last 4 years, with oversight of modernisation of the specialist training program around contemporary models of care.			
DG	Psychological scientist	Educational training in psychology at both undergraduate [BSc (Hons)] and postgraduate (PhD) levels.	15	-	14
		Research portfolio sits at the interface of the psychological and behavioural sciences, and utilises basic and applied research paradigms to advance knowledge and practice on the complexities of human performance and health.			
HS	Clinician Researcher	Roles involve intersection of clinical/teaching and research.	18	40	33
		Postgraduate Masters-level physiotherapy musculoskeletal teaching (including a pain unit). Extensive clinical practice across primary and tertiary care settings including in extended scope roles.			
		Clinical research focused on musculoskeletal health and person-centred pain care; heath systems and services; models of care; systems strengthening; capacity building in health workforce to support improved pain care.			

^a^ 5 years into PhD training.

We utilised a “best fit” framework synthesis (34–36) approach to analyse and develop new insights on the behavioural determinants to the adoption of biopsychosocial musculoskeletal pain care (hereafter, referred to as “adoption”). Framework synthesis offered a theory-based synthesis method, and utility and value have already been demonstrated in areas of healthcare with policy relevance (35). This approach allowed the application of a primarily deductive approach (34, 36) to the data, yet also permitted inductive revision and supplementation of the “a priori” theory (35). The components of the COM-B model, “capability”, “opportunity”, “motivation” and “behaviour”, and the 14 domains of the TDF (23), formed the “a priori” framework for the synthesis. The approach enabled us to further interrogate from a behavioural perspective, previously established themes from our qualitative review and identify gaps in the knowledge. The definition of each COM-B component and TDF domain (Supplementary Material) were provided to research team members to facilitate consistent interpretation and mapping of the data to the framework. The following steps demonstrate our phased methodologic approach:

### Step 1 Mapping previously derived subthemes to the COM-B model and TDF

Three team members (WN, DB, DG) were provided with a Microsoft Excel spreadsheet consisting of the 46 subthemes drawn from our previous qualitative synthesis (15). They independently mapped these subthemes against the COM-B model and TDF. Conflicts or discrepancy in the mapping results were resolved through discussion and consensus with the mapping team and an additional independent team member (HS).

### Step 2 Identifying stakeholders who may potentially influence healthcare professionals' adoption of a biopsychosocial model in musculoskeletal pain care

All team members studied the overall outcomes from Step 1. Based on the extracted data from the studies included in our previous review (15), the team derived a minimum list of stakeholders. This included stakeholders from across the whole-of-health from the micro-level (clinical interface), meso-level (health service provision and workforce training), and macro-level (health system).

In this step, a list of stakeholders and the previously derived 14 main themes were presented as a word document to the team. Members were tasked with independently answering the question “Is the theme critical for this stakeholder group to intervene on to improve biopsychosocial adoption?” We defined “intervene” as “to become intentionally involved in influencing and improving adoption”. More than one stakeholder group could be selected to intervene for each theme, and team members could nominate any other relevant stakeholder group not otherwise mentioned but considered important potential contributors to adoption. Consensus on the most appropriate “proposed stakeholders” to potentially intervene on each of the 14 themes was achieved through a meeting.

### Step 3 Deciding what intervention functions were important in supporting healthcare professionals' adoption of a biopsychosocial model

To establish which category of interventions could potentially shift the behaviour of healthcare professionals to improve adoption, team members were asked to independently respond to this question “From the nine BCW intervention functions (education, persuasion, incentivisation, coercion, training, restriction, environmental restructuring, modelling, enablement), which function(s) meet the affordability, practicability, effectiveness/cost-effectiveness, acceptability, side-effects/safety, equity (APEASE) criteria to improving adoption?” (Definition of these intervention functions are shown later under results). We applied the APEASE criteria (23) to make strategic judgments on what might be the most appropriate intervention(s), with real world applicability. The description of the APEASE criteria can be found in Supplementary Material. Responses were collated and recorded on a Word document.

### Step 4 Interpreting and conceptualising: Formulation of a conceptual model to understand the behavioural determinants and reach consensus on who may potentially influence healthcare professionals' adoption of the biopsychosocial model

A conceptual model, comprised of the pre-determined concepts (from the COM-B model and TDF) and newly-derived concepts integrated together, was developed to describe the behavioural determinants and explained how alignment of the various stakeholder groups could help achieve the goal of biopsychosocial musculoskeletal pain care.

The combination of the “a priori” concepts from the COM-B and TDF, and the research team members' newly-derived concepts from the interpretation of the data, highlighted the use of both deductive and inductive analyses in this step. The resultant synthesis of the conceptual model was built on the COM-B model and TDF, and was further enhanced with additional concepts from our qualitative review (15). This moved the description of the data used for the analysis to a higher level of abstraction and created an integrative conceptual framework. WN conceptualized and drew the conceptual model, the rest of the team commented on and refined the model to accurately reflect a visual representation of the behavioural determinants.

### Step 5 Derivation of a pragmatic framework of behaviour change techniques to improve adoption

A behaviour change technique (BCT) is defined as “an active component of an intervention designed to change behaviour” (23). Here, we were interested to identify the observable, replicable, and irreducible components (i.e., active ingredients) of an intervention (31) that could facilitate behaviour change in healthcare professionals towards improved adoption. To approach this step, we gave thoughtful consideration to a principle used to achieve rigor in qualitative research analysis (37)—a hybrid approach of inductive and deductive analysis (38). Using both inductive and deductive analyses enabled us to collate a more comprehensive list of BCTs, grounded in the evidence-base, that would not have been achieved using either approach alone.

**Figure 2:**
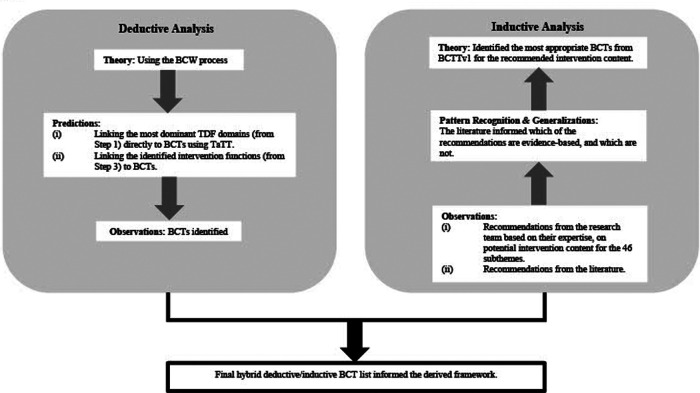
A graphic summary of the process of deductive and inductive analyses applied to derive a hybrid list of BCTs. Deductive analysis involves applying a theory (in this case, the Behaviour Change Wheel) to make predictions to our observed data. Inductive analysis involves observing our data thoroughly using researches’ reflexivity and the evidence-base, to look for patterns, trends and generalizations in the data, to see if the data fits into a suggested theory (i.e., behavioural change techniques). BCTs, behaviour change techniques; BCTTv1, behaviour change techniques taxonomy version 1; BCW, behavioural change wheel; TDF, theoretical domains framework; TaTT, theory and technique tool.

Figure 2 shows a graphic summary of the applied processes of deductive and inductive analyses.

### Deductive analysis

**(i) Linking TDF domains directly to BCTs using the Theory and Technique Tool (TaTT).**

Based on the overall mapping results in Step 1, in order to identify priority areas for focusing intervention efforts to improve training and support for healthcare professionals' pain education, the most dominant TDF domains (as defined by count of the number of subthemes, at the micro-level, meso- and macro-level) populated by the mapping data were identified (WN). The most dominant TDF domains were linked directly to BCTs using the Theory and Technique Tool **(**TaTT) (39) via the Theory and Technique Tool website (40) through deductive inference. On the TaTT, the strength of a particular link between a mechanism of action/TDF domain and a BCT is denoted by four different coloured cells—white (“no evidence”), yellow (“inconclusive”), blue (“non-links”) or green (“links”) (39, 41, 42). For the purpose of this study, we wanted to identify the most effective BCTs to target on the most dominant TDF domains, hence only the green cells (“links”) were considered.

**(ii) Linking intervention functions to BCTs.**

From Step 3, the identified intervention functions were then linked to the BCTs on Behavioural Change Techniques Taxonomy v1 (BCTTv1) (Supplementary Material) deductively by the BCW (23, 31). The links between intervention functions and BCTs had been established by expert consensus (23). To narrow down the list of BCTs for selection, for each intervention function, we only considered the most frequently used BCTs (43).

Using both deductive methods of linking, we were able to make predictions and identify which BCTs could potentially be adopted to target specific behavioural change. At this point, we assessed whether the BCTs selected would fit into proposed intervention(s) that also met the APEASE criteria i.e., were affordable, practical, cost-effective, acceptable, safe and equitable in the real world. One researcher (WN) collated, observed, and compared the two list of BCTs derived from both deductive methods of linking.

### Inductive analysis

Based on their areas of expertise, the research team discussed their recommendations on how to address the target behaviour (Table 2) and suggested potential intervention content. These data were recorded in an Excel spreadsheet and interrogated against the 46 subthemes (the dataset) to ensure recommendations targeted specific areas relevant to healthcare professionals assessing, identifying and analysing biopsychosocial factors contributing to musculoskeletal pain. Recommendations from the team were collaboratively discussed and synthesized inductively and iteratively. The literature was then reviewed to examine if these recommendations were reflected in the evidence-base. The entire inductive analysis process was both iterative and reflexive. The most appropriate BCTs were identified by coding the intervention content to the most relevant grouping and definition of the BCT on the BCTTv1.

**Table 2:** Specifications of the target behaviour.

Target behaviour	Healthcare professionals’ adoption of a biopsychosocial approach in musculoskeletal pain care.
Who	Healthcare professionals licensed to provide musculoskeletal pain care. Examples include (but not restricted to) anaesthetists, chiropractors, clinical psychologists, general practitioners, nurses, occupational therapists, osteopaths, pain physicians, physiotherapists and rheumatologists.
What	Assess, identify and analyse biopsychosocial factors contributing to each individual’s musculoskeletal pain experience.
How	Using authentic communication and critical clinical reasoning, with strong therapeutic alliance.
Whom	In partnership with patients; people with lived pain experience; consumers.
When	During clinical consultation, first, ongoing or review appointments.
Where	In clinical practice settings.

### Derivation of a final framework of BCTs

Based on what is in common between the BCTs derived from deductive linking, and the BCTs derived from inductive analysis of recommendations from the research team (that is also evidence-based), a hybrid list of BCTs (required at the bare minimal) to facilitate change towards improved adoption were identified.

The recommended strategies that could potentially empower healthcare professionals to assess, identify and analyse biopsychosocial factors contributing to musculoskeletal pain were collated and reported under “discussion”. The research team also derived a worked example of how to use BCTs to improve healthcare professionals' adoption of a biopsychosocial approach across the whole-of-health.

## Results

### Step 1 Mapping previously derived subthemes to the COM-B model and TDF

Table 3 provides a detailed breakdown of how the subthemes from our previous qualitative evidence synthesis (15) mapped onto the COM-B model and TDF. A subtheme could be mapped onto more than one component on the COM-B and more than one domain on the TDF. An overview of the number of subthemes mapped to each COM-B component and TDF domain is provided in Supplementary Table S5. Overall, the 46 subthemes (barriers and enablers to the adoption of a biopsychosocial approach) mapped on 5/6 components on the COM-B model (with the exception of physical capability), and 12/15 domains on the TDF (with the exception of physical skills, optimism and goals), reflecting that the adoption of a biopsychosocial approach involved a complex set of behavioural determinants across multi-levels of healthcare.

**Table 3:** Healthcare professionals’ barriers and enablers to the biopsychosocial approach mapped onto the COM-B model and TDF.

Themes	Subthemes	COM-B domains	TDF domains
Micro level			
**1.1 Healthcare professional knowledge and skills** Healthcare professional’s knowledge of psychosocial factors, evidence-based practice and other healthcare disciplines, and their interpersonal and people skills.	1.1.1 Healthcare professionals are aware of the importance of psychosocial factors, but are vague about what those factors are.	Psychological capability	Knowledge
	1.1.2 Lack of knowledge of the levels of evidence & the concept of evidence-based practice.		Knowledge
	1.1.3 The knowledge (or the lack of knowledge) on how to identify psychosocial factors (including the use of questionnaires and instruments for screening); manage psychosocial factors or yellow flags; or the lack of ability to apply the biopsychosocial model.		Knowledge Skills
	1.1.4 Healthcare professionals’ default approach of addressing “biomedical” or “red flags” first (or only), then “psychosocial” or “yellow flags” or “biopsychosocial”.	Psychological capability Reflective motivation	Memory, attention and decision processes
	1.1.5 The ability (or inability) of the healthcare professionals to manage the clinician-patient alliance.	Psychological capability	Skills
	1.1.6 The ability (or inability) to use communication and interpersonal skills (e.g. counselling, explaining, instructing, listening, reassuring, motivating, promoting and selling a management approach).		Skills
	1.1.7 The knowledge (or the lack of knowledge) of what other healthcare professionals do, other treatment options, when and where to refer to.		Knowledge
	1.1.8 The skill (or the lack of skill) to manage and negotiate health beliefs and patients’ expectations.		Skills
	1.1.9 The skill (or the lack of skill) to manage patients’ emotions and reactions.		Skills
	1.1.10 The knowledge of individualized or personalized care.		Knowledge
	1.1.11 The knowledge that to treat chronic pain, it is not about curing it; rather, managing pain.		Knowledge
	1.1.12 The knowledge that the pain score is a means for the patient to communicate a more general suffering; & the skill to identify & modify pain, specific to patients’ aggravating activity or affected behaviour.		Knowledge Skills
**1.2 Healthcare professional personal factors** Individual factors and arbitrary choices of healthcare professionals: their emotions associated with chronic pain management; beliefs; level of self-awareness with pre-existing clinical habits; desire to learn; the role and professional identity they assumed; qualifications and work experience associated with the use of a biopsychosocial approach in pain care.	1.2.1 Healthcare professionals’ negative emotions associated with the management of chronic pain, psychosocial factors & the use of CPGs & questionnaires.	Automatic motivation	Emotion
	1.2.2 Healthcare professionals may have habits which they subconsciously continue with; or they may consciously not feel a desire to learn; or they may be self-aware, with an ability to reflect on evidence and clinical experience.	Automatic motivation Reflective motivation	Behavioural regulation Intentions Memory, attention and decision processes Reinforcement
	1.2.3 Healthcare professionals consider OR don't consider it their role (including the role to refer on) & scope of practice to use the BPS approach or follow BPS oriented guidelines.	Reflective motivation	Professional role and identity
	1.2.4 Healthcare professionals biomedical or biopsychosocial treatment orientation or professional identity.		
	1.2.5 Healthcare professionals helpful OR unhelpful beliefs (including misconceptions) towards the use of a BPS approach or the use of guidelines.		Beliefs about consequences
	1.2.6 Healthcare professionals additional qualifications & relevant work experience associated with the use of a BPS approach.	Psychological capability Reflective motivation	Knowledge Skills Professional role and identity
**1.3 Healthcare professional misconceptions of clinical practice guidelines (CPGs)** Healthcare professionals may misunderstand guidelines as being too generic, simplistic, prescriptive or lacking in flexibility to account for the necessary individualised management of musculoskeletal pain. The presentation of information on guidelines may be unappealing to learning quickly.	1.3.1 Guideline recommendation(s) perceived as uncertain OR unhelpful.	Reflective motivation	Beliefs about consequences
	1.3.2 Guidelines are perceived as generic OR simplistic OR too mechanistic, prescriptive OR rigid in the management of patients’ musculoskeletal conditions.		
	1.3.3 Guidelines are perceived as not providing adequate clinical tools OR perceived as having too many psychosocial questionnaires to choose from.		
	1.3.4 Healthcare professionals are generally not inclined to pay attention to CPGs, the presentation of CPGs is not appealing and may be incompatible with healthcare professionals’ way of learning.		Memory, attention and decision processes
	1.3.5 Healthcare professionals are unclear of what “non-specific” means in the non-specific musculoskeletal pain diagnosis in CPGs.	Psychological capability	Knowledge
**1.4 Healthcare professional perceptions about patient factors** Healthcare professional perceptions and judgments about patient factors may overemphasize the psychological framing of the condition and the negative stereotype of the difficult patient.	1.4.1 Healthcare professionals’ interpretation or judgment of patients’ lack of motivation or ulterior motives.	Reflective motivation	Intentions
	1.4.2 Patients’ biomedical focus or expectations, unhelpful beliefs and attitudes and poor health literacy can impact on their care and clinical management.	Social opportunity	Social influences
	1.4.3 Healthcare professionals’ judgments about patients’ circumstances, including the judgment of patients’ social issues & involvement with a legal case, which may overemphasize the negative stereotype of chronic musculoskeletal pain.	Reflective motivation	Intentions Beliefs about consequences
	1.4.4 Patients’ complexity of clinical presentation prompts the exploration of psychosocial factors or the use of recommendations from CPGs.	Psychological capability	Skills Memory, attention and decision processes
**1.5 Healthcare professional perception of time** Healthcare professionals perceived there is insufficient time to explore psychosocial factors within a clinical consultation, and the lack of time for learning.	1.5.1 Healthcare professionals perceived that there is insufficient time to explore psychosocial factors within a clinical consultation, and no time to reflect, or read and learn about CPGs.	Physical opportunity Reflective motivation	Environmental context and resources
Meso level			
**2.1 CPG formulation** Guideline development may be unable to account for different categories of patients, patients’ expectations, healthcare professionals’ former knowledge and training, contextual factors and real-world situations.	2.1.1 Guideline care may not be compatible with the concept of delivering individualized care.	Psychological capability Social opportunity	Knowledge Social influences
	2.1.2 Compatibility of guideline care to healthcare professionals’ clinical practice, former knowledge, training, and real-world practice.	Reflective motivation Social opportunity	Beliefs about capabilities Social influences
	2.1.3 The existence of CPGs help to facilitate and coordinate teamwork among healthcare professionals, provided healthcare professionals are familiar with the content.	Social opportunity	Social influences
	2.1.4 Guidelines are a good source of information to patients and contribute to their understanding of evidence-based treatment options.		
	2.1.5 Guidelines provide up-to-date, useful information and decisional algorithms to help healthcare professionals in their clinical decision making and navigate clinical uncertainty.		
**2.2 Clinical community factors** Ready access and availability of an egalitarian interdisciplinary or multidisciplinary team to consult for challenging clinical cases, and whether or not the treatment orientation and communication among professionals within a team is aligned.	2.2.1 Access & availability (or lack thereof) of a clinical support system or network with an efficient communication channel.	Physical opportunity Social opportunity	Environmental context and resources Social influences
	2.2.2 Conflict or alignment between healthcare professionals in the interpretation about what care is required.	Social opportunity Reflective motivation	Social influences Beliefs about consequences
**2.3 Funding models** Financial barriers such as patients’ lack of health insurance, the lack of funding to incentivise healthcare professionals for their time, effort and qualifications, as well as the funding required to construct models of care appropriate to deliver high value musculoskeletal pain care may impact the feasibility of using the biopsychosocial approach.	2.3.1 The funding model used (i.e. government group insurance, private healthcare insurance, workers’ compensation board, individual out-of-pocket expenses) and the financial feasibility of the BPS approach can encourage or discourage the use of the approach.	Physical opportunity	Environmental context and resources Reinforcement
**2.4 Health service provision** Work processes such as needing to complete a large amount of administrative work, or performance indicators such as requiring to see many patients or the structure of group therapy sessions may not facilitate the use of a biopsychosocial approach to pain care.	2.4.1 The level of alignment of work processes within organizations to evidence-based methods, or a BPS approach.	Physical opportunity Social opportunity	Environmental context and resources Social influences
**2.5 Resourcing issues** Lack of resources such as time, specialist services, appointment slots and clinic infrastructure to support the use of a biopsychosocial approach to pain care.	2.5.1 Insufficient time and frequency of consultation, and too much time on long waitlist for referrals to specialist services and investigations are resource-related time barriers to the use of a BPS approach.	Physical opportunity	Environmental context and resources
	2.5.2 The availability (or the lack of) of specialist services, appointment slots, clinic infrastructure and resources (such as educational content and tools) to support a BPS approach.		
**2.6 Workforce training issues** Workforce training issues such as a lack of explicit communication training, counselling and psychosocial competencies in undergraduate and postgraduate training programs.	2.6.1 Lack of counselling/psychosocial training to help healthcare professionals apply a BPS approach.	Physical opportunity	Environmental context and resources
Macro-level			
**3.1 Health policy** Health policy may not prioritise or align to best practice, evidence-based care of musculoskeletal conditions.	3.1.1 The level of political support or attention provided by governments, compensable bodies, professional associations and regulatory boards to provide evidence-based care.	Physical opportunity Social opportunity	Environmental context and resources Social influences
**3.2 Organizational factors** Organizational factors such as healthcare financing models and regulations within healthcare delivery may not align with high value, person-centred musculoskeletal pain care.	3.2.1 Criterion for the funding set by healthcare systems, insurers or organizations can be compatible or incompatible with the use of a BPS approach.	Physical opportunity Social opportunity	Environmental context and resources Social influences
	3.2.2 Regulations within healthcare systems or workplace culture may promote or obstruct the use of a BPS approach.		
**3.3 Social factors** Social factors such as the persistence and dominance of the biomedical paradigm in healthcare professions and systems, and stigma towards psychological services.	3.3.1 The persistence of a biomedical culture in healthcare professions & systems.	Social opportunity	Social influences
	3.3.2 Social stigma towards psychological services.		
	3.3.3 The pervasiveness of information spread via mass media may not be aligned to a BPS model of care.		

BPS, biopsychosocial; COM-B, Capability Opportunity Motivation-Behaviour; CPGs, Clinical Practice Guidelines; TDF, Theoretical Domains Framework.

### Step 2 Identifying stakeholders who may potentially influence healthcare professionals’ adoption of a biopsychosocial model in musculoskeletal pain care

Table 4 shows the tabulation of the identified key stakeholder groups. Healthcare professionals, educators, guideline developers, workplace managers, and policymakers were the stakeholder groups identified as target audiences for potential interventions. Researchers were considered as relevant to all five stakeholder groups. Researchers' roles may involve an investigation into healthcare professionals' behaviour, educators' behaviour, evaluation of clinical practice guideline implementation, workplace programs or policy implementation.

**Table 4:** The key stakeholder groups to target behavioural interventions for the respective barriers and enablers to the adoption of the biopsychosocial approach.

Themes	Healthcare professionals^a^ including researchers	Educators^b^ including researchers	Guideline developers^c^ including researchers	Workplace managers^d^ including researchers	Policymakers^e^ including researchers
Micro-level	√	√			
**1.1 Healthcare professional knowledge and skills** Healthcare professional’s knowledge of psychosocial factors, evidence-based practice and other healthcare disciplines, and their interpersonal and people skills.					
**1.2 Healthcare professional personal factors** Individual factors and arbitrary choices of healthcare professionals: their emotions associated with chronic pain management; beliefs; level of self-awareness with pre-existing clinical habits; desire to learn; the role and professional identity they assumed; qualifications and work experience associated with the use of a biopsychosocial approach in pain care.	√	√			
**1.3 Healthcare professional misconceptions of clinical practice guidelines (CPGs)** Healthcare professionals may misunderstand guidelines as being too generic, simplistic, prescriptive or lacking in flexibility to account for the necessary individualised management of musculoskeletal pain. The presentation of information on guidelines may be unappealing to learning quickly.	√	√	√		
**1.4 Healthcare professional perceptions about patient factors** Healthcare professional perceptions and judgments about patient factors may overemphasize the psychological framing of the condition and the negative stereotype of the difficult patient.	√	√			
**1.5 Healthcare professional perception of time** Healthcare professionals perceived there is insufficient time to explore psychosocial factors within a clinical consultation, and the lack of time for learning.	√			√	
**Meso-level**			√		
**2.1 CPG formulation** Guideline development may be unable to account for different categories of patients, patients’ expectations, healthcare professionals’ former knowledge and training, contextual factors and real-world situations.					
**2.2 Clinical community factors** Ready access and availability of an egalitarian interdisciplinary or multidisciplinary team to consult for challenging clinical cases, and whether or not the treatment orientation and communication among professionals within a team is aligned.	√			√	
**2.3 Funding models** Financial barriers such as patients’ lack of health insurance, the lack of funding to incentivise healthcare professionals for their time, effort and qualifications, as well as the funding required to construct models of care appropriate to deliver high value musculoskeletal pain care may impact the feasibility of using the biopsychosocial approach.				√	√
**2.4 Health service provision** Work processes such as needing to complete a large amount of administrative work, or performance indicators such as requiring to see many patients or the structure of group therapy sessions may not facilitate the use of a biopsychosocial approach to pain care.	√			√	√
**2.5 Resourcing issues** Lack of resources such as time, specialist services, appointment slots and clinic infrastructure to support the use of a biopsychosocial approach to pain care.				√	√
**2.6 Workforce training issues** Workforce training issues such as a lack of explicit communication training, counselling and psychosocial competencies in undergraduate and postgraduate training programs.		√		√	√
Macro-level					√
**3.1 Health policy** Health policy may not prioritise or align to best practice, evidence-based care of musculoskeletal conditions.					
**3.2 Organizational factors** Organizational factors such as healthcare financing models and regulations within healthcare delivery may not align with high value, person-centred musculoskeletal pain care.				√	√
**3.3 Social factors** Social factors such as the persistence and dominance of the biomedical paradigm in healthcare professions and systems, and stigma towards psychological services.	√	√		√	√

CPG, clinical practice guidelines; √, represents consensus has been achieved among research team members when asked the question “Is the theme critical for the stakeholder group to intervene on to improve biopsychosocial adoption?”.

^a^ Medical or allied health professionals licensed to provide musculoskeletal pain care and deliver health care services to patients. Examples include (but not restricted to) anaesthetists, chiropractors, clinical psychologists, general practitioners, nurses, occupational therapists, osteopaths, pain physicians, physiotherapists and rheumatologists.

^b^ Teachers who provide education, instruction or clinical guidance in musculoskeletal sciences and/or pain curriculums, in the capacity as college/university educators, tutors, clinical educators and/or facilitators of continuing professional education.

^c^ Researchers, professional organizations/associations, or department/ministry of health who develop clinical practice guidelines to grade evidence and develop recommendations based on best available evidence for musculoskeletal pain conditions.

^d^ Clinic managers who oversee the day-to-day operation or management of healthcare facilities/musculoskeletal outpatient clinics, maintain responsibility for the administrative aspects of the clinical services, and liaise between healthcare professionals and patients.

^e^ Members of professional organizations/associations, department/ministry of health or other government departments who are involved in legislation and healthcare funding rules, and are responsible for formulating healthcare policies and making policy decisions.

### Step 3 Deciding what intervention functions were important in supporting healthcare professionals’ adoption of a biopsychosocial model

Table 5 provides a list of targeted intervention functions that could help to address specific barriers to healthcare professionals' adoption of a biopsychosocial approach to musculoskeletal pain care. A supporting rationale is shown. This is based upon considering criteria such as affordability, practicability, effectiveness/cost-effectiveness, acceptability, side-effects/safety, and equity (APEASE). Team members discussed and reached consensus that the essential intervention functions important in supporting healthcare professionals' adoption of a biopsychosocial model were education, training, environmental restructuring, modelling and enablement.

**Table 5:** Selection of the intervention functions and rationale based on the APEASE criteria.

Intervention functions	Definition	Does the intervention function meet the APEASE criteria?
**Education**	Increasing knowledge or understanding.	Yes. Education is an essential tool that can be used to create the awareness, change knowledge, attitudes and beliefs of healthcare professionals. It is suggested the design of a pain curriculum be considered in the context of affordability, length of time it takes to upskill healthcare professionals and the cost-effectiveness of the program.
**Persuasion**	Using communication to induce positive or negative feelings or stimulate action.	As a standalone intervention, may be ineffective or minimally effective as there is evidence from our previous study (15) that healthcare professionals are aware of the biopsychosocial approach to musculoskeletal pain care, yet they lack the confidence and capability to apply it in clinical practice.
**Incentivisation**	Creating an expectation of reward.	Challenges acceptability, as adoption of a biopsychosocial approach to pain care is a best practice standard. Using social rewards or professional accolades to recognize individuals or clinics or organisations for implementing biopsychosocial pain care may be an appropriate incentive (versus monetary gains).
**Coercion**	Creating an expectation of punishment or cost.	Unacceptable and unethical to healthcare professionals.
**Training**	Imparting skills.	Yes, ongoing training can be embedded within the continuing professional development requirement to maintain recency of practice and reflect alignment with evidence and best practice standards.
**Restriction**	Using rules to reduce the opportunity to engage in the target behaviour (or to increase the target behaviour by reducing the opportunity to engage in competing behaviours).	Impractical, as there are no options to restrict in this context.
**Environmental restructuring**	Changing the physical or social context.	Yes. Use of virtual “community of practice” can mitigate against geographical barriers to help foster shared learning and useful discussion among healthcare professionals to support the adoption of biopsychosocial musculoskeletal pain care. Project ECHO (44) is an example of a collaborative model/virtual community that provides access to knowledge, mentorship and ongoing support for healthcare professionals.
**Modelling**	Providing an example for people to aspire to or imitate.	Yes. Support and leadership from opinion leaders, clinical champions, and patient advocates with lived experience, in the field of musculoskeletal pain, are helpful.
**Enablement**	Increasing means/reducing barriers to increase capability (beyond education and training) or opportunity (beyond environmental restructuring).	Yes. Data registries, such as the electronic Persistent Pain Outcomes Collaboration (ePPOC), facilitate the collection of data from pain management services. This helps to analyse healthcare utilization and outcomes and these data can be used for benchmarking practice and to promote research into important areas of pain management (45). Websites such as the Cochrane musculoskeletal group (46) and the International Association of the Study of Pain (IASP) (47) are helpful online platforms that collate the latest scientific evidence and enable sharing of these trustworthy information to healthcare professionals and patients to inform clinical decision making.
**Suggested intervention functions**	**Education** **Training** **Environmental restructuring** **Modelling** **Enablement**	

APEASE, affordability, practicability, effectiveness/cost-effectiveness, acceptability, side-effects/safety, and equity.

It is important to note that in this step, applying the APEASE criteria to decide on the intervention functions is essentially a judgment call by the research team, based on our researchers' reflexivity and positionality (Table 1). Our assessment using the APEASE criteria may or may not accurately represent the views of stakeholder groups such as the workplace manager and policymaker, as there is no such representation within the research team. However, it is noteworthy that two of the team members (DB, HS) have relevant experience in collaborating with service providers, workplace managers and policymakers in their clinical and research scope of work. Whether the selected intervention functions will result in improved adoption of biopsychosocial musculoskeletal pain care remains to be tested.

### Step 4 Interpreting and conceptualising: formulation of a conceptual model to understand the behavioural determinants, and reach consensus on who may potentially influence healthcare professionals’ adoption of the biopsychosocial model

**Figure 3:**
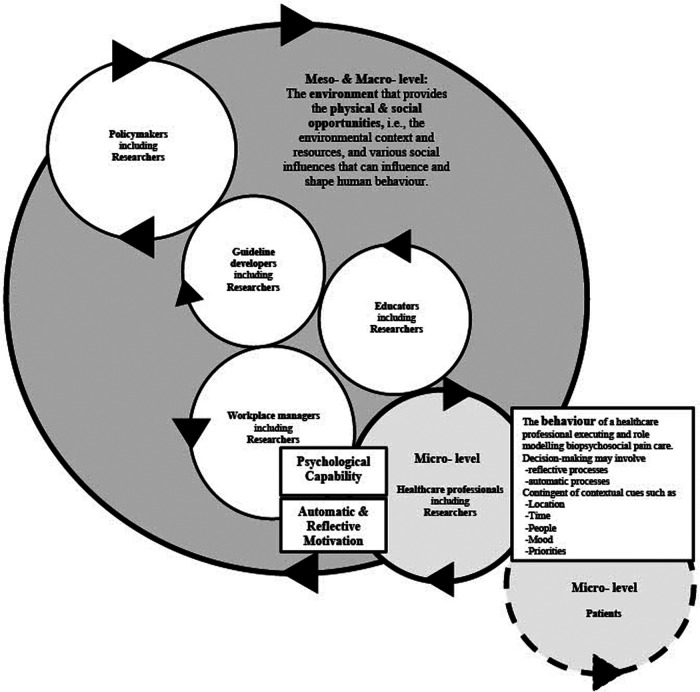
A conceptual cogwheel model outlinning the behavioural determinates of healthcare professionals adopting biopsychosocial pain care, and aligning stakeholders towards enacting emergent, novel behaviours supporting pain care. The terms “psychological capability”, “automatic & reflective motivation”, “behaviour”, “physical & social opportunity” are concepts from the COM-B model. The terms “enviromental context and resources” “social influences” are concepts from the TDF. COM-B, capability opportunity motivation-behaviour model; TDF, theoretical domains framework.

A conceptual model is shown to simplify the behavioural determinants of healthcare professionals adopting biopsychosocial pain care. The model aligns stakeholders towards enacting emergent, novel behaviours supporting biopsychosocial pain care. Figure 3 provides readers with a summary at one glance to understand the behavioural determinants and the major stakeholder groups that need to be involved, to help support healthcare professionals to achieve biopsychosocial pain care.

A new conceptual model differentiated from the original COM-B model (23) was developed (Figure 3) and demonstrates the relationship between “capability”, “motivation”, “behaviour”, and “opportunity”. Unlike the original COM-B model that does not give dominance to either factor “capability”, “opportunity”, or “motivation” influencing behaviour, this graphic proposes the environment (i.e., physical and social opportunity) in which healthcare professionals practise is crucial to adoption (illustrated as the big shaded circle, comprising of other stakeholder groups). Healthcare professionals (illustrated as the smaller circle) are surrounded by environmental context and social influences (physical and social opportunities) of the healthcare system, which will influence and shape how they behave. However, behaviours may sometimes be “out of context”- that is why the smaller circle representing the healthcare professional is drawn slightly out of the big circle representing the environmental context. Healthcare professionals' behaviours require psychological capability in decision-making in complex and varied clinical scenarios and may involve both reflective and automatic processes (or motivation) (48). Reflective processes refer to the cognitive ability, awareness and conscious deliberation to make complex clinical decisions before enacting behaviour; whereas automatic processes are learnt predispositions/proclivity to think or act in a given way or habits. These processes are cued by external factors (location, time, or people) or internal reactions and factors (mood or priorities) of the healthcare professional (48, 49).

A hypothetical patient cogwheel (illustrated as the circle with dotted line) is interacting with and influencing the behaviour of the healthcare professional, and vice versa. Every stakeholder, illustrated as interconnected individual circles, plays a role in the whole healthcare landscape (illustrated as the big shaded circle, i.e., representing the environmental context and social influences). The behaviour of a complex system emerges from the interaction of the six stakeholder groups (patients, healthcare professionals, educators, workplace managers, guideline developers and policymakers) (50), and will potentially influence and change the environmental and social context towards supporting the goal of biopsychosocial pain care, with the whole being greater than the sum of its parts. In other words, these emergent, novel behaviours extend beyond the clinician-patient system to the remainder of the healthcare system, encouraging communication and relationship-centered care among stakeholders across all levels of healthcare (51, 52). A circle coming in contact with another circle here represents the interdependency of the stakeholders to one another, though alignment of the stakeholders is not necessarily mutually exclusive (e.g., workplace managers may come directly in contact with policymakers). Arrows are used to denote the dynamicity of the system, i.e., moving one cogwheel can potentially influence and impact the adjacent aligning cogwheel. Subsequently, the interdependencies can set the whole cogwheel system in motion (synonymous to cooperation and collaboration between stakeholders). However, if one cogwheel moves in a direction that does not align with the rest of the cogwheels, it may potentially create a barrier or “logjam” in the system.

### Step 5 Derivation of a pragmatic framework of behaviour change techniques to improve adoption

As shown in Table 3 (and Supplementary Table S5), the majority of the micro-level subthemes mapped to the “knowledge” and “cognitive and interpersonal skills” domain on the TDF, whereas the majority of the meso-level and macro-level subthemes mapped to the “environmental context and resources” and “social influences” domain on the TDF.

Table 6 shows the list of BCTs identified via two deductive methods of linking, inductive analysis and the final hybrid list of BCTs identified by both deductive linking and inductive analysis. The full BCT taxonomy v1 and the definition of each BCT are provided in Supplementary Table S4.

**Table 6:** Identified behaviour change techniques derived from deductive linking, inductive analysis resulting in the final hybrid list.

	Deductive Linking/Analysis			
	(i) Dominant TDF domain	Links to BCTs identified on TaTT as “green” links	(ii) Identified intervention functions	Most frequently used BCTs (from BCTTv1) for specific intervention function
**Micro-level**	**Knowledge**	2.6 Biofeedback 4.1 Instruction on how to perform behaviour 4.2 Information about antecedents 5.1 Information about health consequences 5.3 Information about social and environmental consequences	**Education**	2.2 Feedback on behaviour 2.3 Self-monitoring of behaviour 2.7 Feedback on outcome(s) of behaviour 5.1 Information about health consequences 5.3 Information about social and environmental consequences 7.1 Prompts/cues
	**Cognitive and interpersonal skills**	4.1 Instruction on how to perform behaviour 8.1 Behavioural practice/rehearsal 8.7 Graded tasks	**Training**	2.2 Feedback on behaviour 2.3 Self-monitoring of behaviour 2.7 Feedback on outcome(s) of behaviour 4.1 Instruction on how to perform the behaviour 6.1 Demonstration of the behaviour 8.1 Behavioural practice/rehearsal
**Meso- & Macro-level**	**Environmental context and resources**	3.2 Social support (practical) 7.1 Prompts/cues 7.5 Remove aversive stimulus 12.1 Restructuring the physical environment 12.2 Restructuring the social environment 12.3 Avoidance/reducing exposure to cues for the behaviour 12.5 Adding objects to the environment	**Environmental restructuring**	7.1 Prompts/cues 12.1 Restructuring the physical environment 12.5 Adding objects to the environment
	**Social influences**	3.1 Social support (unspecified) 3.2 Social support (practical) 6.2 Social comparison 6.3 Information about others’ approval 10.4 Social reward	**Modelling**	6.1 Demonstration of the behaviour
			**Enablement**	1.1 Goal setting (behaviour) 1.2 Problem solving 1.3 Goal setting (outcome) 1.4 Action planning 1.5 Review behaviour goal(s) 1.7 Review outcome goal(s) 2.3 Self-monitoring of behaviour 3.1 Social support (unspecified) 3.2 Social support (practical) 12.1 Restructuring the physical environment 12.5 Adding objects to the environment
**Inductive Analysis** (2 examples provided)				
**Subtheme**	**Example of intervention content**			**Identified BCT (from BCTTv1)**
1.1.4 Healthcare professionals’ prioritizes addressing “biomedical” or “red flags” first (or only), then “psychosocial” or “yellow flags” or “biopsychosocial”.	Introduce a checkbox on the initial assessment form to prompt for a psychosocial assessment with the use of questionnaires such as the Örebro Musculoskeletal Pain Questionnaire (ÖMPQ).			7.1 Prompts/cues
1.1.5 The ability (or inability) of the healthcare professionals to manage the clinician-patient alliance.	Training in the form of practice and empathetic reflective feedback from clinician to patient enhances overall communication style and patient-centred communication behaviours.			8.1 Behavioural practice/rehearsal
**Final hybrid list of BCTs**				
**Based on both deductive and inductive analysis, the most relevant BCTs required at the bare minimum to facilitate change towards improved adoption of the biopsychosocial approach:**				
Micro-level				
4.1 Instruction on how to perform a behaviour				
8.1 Behavioural practice/rehearsal				
Meso- and Macro-level				
3.1 Social support (unspecified)				
3.2 Social support (practical)				
7.1 Prompts/cues				
12.1 Restructuring the physical environment				

BCTs, behaviour change techniques; BCTTv1, behaviour change techniques taxonomy version 1; TDF, theoretical domains framework; TaTT, theory and technique tool. BCTs underlined are in common, using the two deductive methods of linking.

Overall, six BCTs from BCTTv1 were identified as relevant and the minimum required to facilitate healthcare professionals' behaviour change towards improved adoption. At the micro-level, BCTs “4.1 instruction on how to perform a behaviour” and “8.1 behavioural practice/rehearsal” were relevant. At the meso- and macro-level, BCTs “3.1 social support (unspecified)”, “3.2 social support (practical)”, “7.1 prompts/cues”, and “12.1 restructuring the physical environment” were relevant.

A template of our synthesized framework of BCTs, with the links between the dominant COM-B components and TDF domains, intervention functions and the selected BCTs as informed by the BCW process (23, 24, 31, 40–43) can be found in Supplementary Table S6.

## Discussion

This study describes a novel systematic approach in which we leveraged principles from the BCW process to (i) identify behavioural determinants that can support the adoption of a biopsychosocial approach, (ii) formulate a novel conceptual model outlining these behavioural determinants, and (iii) derive a framework of BCTs that have the potential to facilitate and improve healthcare professionals' adoption of a biopsychosocial approach to musculoskeletal pain care. This approach provides a blueprint to road test how target interventions can help improve healthcare professionals' understanding of pain by addressing important target behaviours that underpin quality pain care.

In line with the aim of our work, a recent review (26) also investigated and mapped the barriers and facilitators to a biopsychosocial approach against the TDF and subsequently to the TaTT. These colleagues identified 10 TDF domains and 33 BCTs that could foster implementation. Consistent with our findings, the authors highlighted that implementation of a biopsychosocial approach is complex (26). That study also used deductive coding and analysis, and their results were specific to physiotherapy practice. In contrast, by taking a more in-depth and broader whole-of-system approach to driving adoption, our study value-adds to the evidence base by (i) amalgamating the determinants to derive a cogwheel model to enhance understanding of the subject matter from a behavioural perspective, (ii) identifying relevant stakeholder groups to intervene, (iii) suggesting targeted intervention functions and content, and (iv) identifying core BCTs to improve adoption.

The use of both deductive and inductive analysis in our study is a strength of this study as we combined the use of theory with clinical and research expertise. It is important to note the BCW process is not a panacea for behaviour change but a system of using best available evidence, informed judgment and resources to arrive at a strategy to address a specified behaviour (23). Hence, this discussion is structured to elucidate our reasoning processes. Stakeholders working within the health services and systems level may derive practical, useful and actionable insights from our findings.

### Strengths

The conceptual model developed in our study capture a broad system overview on factors and key stakeholders who can potentially influence the adoption of a biopsychosocial approach to musculoskeletal pain care. This cogwheel model reflects a whole-of-system approach and highlight opportunities for behavioural intervention designers and policymakers to target specific initiatives to promote and support and strengthen a system-wide approach to biopsychosocial musculoskeletal pain care. Developing the model from existing evidence-based behaviour change theoretical foundations (23, 24, 27, 31) is also a strength. Our method of conceptual model development is explicit and transparent, allowing readers to see clearly how data from our previous review (15) mapped to the COM-B model and TDF, and how these data are then translated back to the COM-B model to derive the new conceptual model (53). Our constructivist epistemological position towards knowledge construction and the hybrid approach of using both deductive and inductive analysis demonstrate theoretical rigor by accounting for sound and logical reasoning in the analysis process. We incorporated team members' subjective interpretation of the data from various experiential levels of the health system and ensured knowledge generated by this research is usable in real-world healthcare settings (37).

### Limitations

The consensus reached in the team was driven by the knowledge and experience of a small group of clinicians and researchers working in musculoskeletal pain, the majority of whom are clinical and research physiotherapists (Table 1). Physiotherapists develop, maintain and restore maximum movement and functional ability in people and maximise their quality of life by looking at physical, psychological, emotional and social wellbeing, mainly using non-invasive, physical treatments or modalities such as exercises, manual therapy and education (54). High-value, biopsychosocial musculoskeletal pain care encompasses a mixture of conservative, non-invasive treatment methods, education, psychological therapies, pharmacological treatment and only in relevant cases, surgical treatment (55, 56). As such, the research team was unable to offer comprehensive representation of the views of all other healthcare professionals utilising assessment and treatment methods that were also evidence-based, when we came up with the recommendations to address the target behaviour during the inductive analysis at Step 5. The BCTs framework and worked example (see below) have been designed with the purpose of offering some proposed interventions with universal applicability across implementation contexts. However, the BCTs suggested are by no means exhaustive. This does not mean other BCTs are unimportant or ineffective, rather our selection of the BCTs is targeted at addressing the specified healthcare professionals' behaviours at the micro-level, and supporting these behaviours at the meso- and macro-level. We acknowledge the need to externally test and validate the conceptual model and synthesized framework of BCTs we have derived, to assess readiness to change, and to mindfully consider cultural factors influencing clinical community collaboration in different jurisdictions. We also speculate that the barriers to the adoption of a biopsychosocial approach may be “musculoskeletal pain”-agnostic, but since our search criterion for the initial review (15) is limited to musculoskeletal pain conditions, we could not generalise the findings beyond musculoskeletal pain.

### Context is key in influencing healthcare professionals' behaviour towards improved adoption of a biopsychosocial approach to pain care

Data from our previous qualitative review (15) were found to support almost all the constructs in the COM-B model and TDF, with no data not fitting within the “a priori” framework. Consequently, no secondary thematic analysis was required in the “best fit” framework synthesis. It was worth highlighting that none of the coding undertaken in the meta-synthesis process for the previous review (15) was structured explicitly around concepts in the COM-B model and TDF. This implied that our chosen theory was sufficiently broad and a good fit to capture the data. In this current synthesis, the “best fit” method not only tested the theory, i.e., alignment with the COM-B model and TDF, but also supplemented the foundational theory of the COM-B model. The original COM-B model accorded equal value and importance to “capability”, “opportunity” and “motivation” as influencing behaviours (23). The focal point of our novel synthesized conceptual model was “opportunity” (also known as the “environmental context”), appearing as a key target in influencing, shaping and regulating healthcare professionals' behaviour towards improved adoption. Consistent with previous studies (16, 57), context was key in the acquisition of professional knowledge and clinical skills in the learning of the biopsychosocial approach to pain, and it could either enable or hinder learning and practice behaviours.

### Rationale for the selection of BCTs at the micro-level

In order to improve healthcare professionals' pain education training, our findings suggest that we need to prioritize intervention efforts at “knowledge” and “cognitive and interpersonal skills” and target the micro-level (clinical interface). Review-level evidence indicates that existing healthcare professionals' communication skills training uses a combination of information (delivered in the form of written instructions, didactic lectures, on-line learning or clinical tools), verbal or video feedback, modelling, problem-based learning, and practice (58, 59). More broadly, Cochrane reviews have stated that interventions such as education meetings (60), as well as printed educational materials (61), when used alone or combined with other interventions, can be effective to improve healthcare professionals' practice behaviours, but with small effect sizes. Educational meetings alone do not necessarily translate to changing healthcare professionals' ingrained practice behaviour and improved patients' outcomes (17, 20). To increase effectiveness, considerations are therefore needed in the design of education to use interactive, combined with didactic formats (60). To improve the fidelity of education/training interventions, incorporating the following BCTs; “4.1 instruction on how to perform the behaviour”, and “8.1 behavioural practice/rehearsal” into training may be beneficial.

### Rationale for the selection of BCTs at the meso- and macro-level

In order to adequately support pain education for healthcare professionals, our findings suggest a crucial need for targeted intervention efforts at the meso- (health services and workforce training) and macro-level (systems/policy), specifically for TDF domains “environmental context and resources” and “social influences”. Here, aligning implementation efforts of biopsychosocial pain care to the health services and system levels is paramount. As a first step, addressing how clinical communities and the lived environment is structured to modify or create new knowledge, clinical practice guidelines, health services and policy is key. Target levers to support implementation include: establishing strong multi-sectoral partnerships and advocacy across clinical communities, people with lived experience of pain, work and professional organisations, universities, funding and insurance agencies and governments. This can strengthen health systems to support high value musculoskeletal pain care (62). Examples of existing partnerships and collaboration may include: partnering with patient advocates from the Global Alliance of Partners for Pain Advocacy (GAPPA) task force (63) or people with lived experience of pain to create better outcomes in the understanding, research, teaching and management of musculoskeletal pain (64–66); partnering with consumer representatives from Cochrane musculoskeletal review group to develop meaningful and person-centred clinical practice guidelines (46); delivering biopsychosocial-informed education to promote improvements in insurance workers’ pain beliefs and helpful claims management behaviour (67); aligning country-level strategies to address the burden of pain to the newly developed global blueprint/framework for musculoskeletal health (68, 69). Additionally, as highlighted by our previous qualitative review (15), there is a critical need within health systems to support interdisciplinary or multidisciplinary care, especially for complex and chronic pain presentations. Appropriate funding or a reorientation of funding to develop models of care to deliver high value musculoskeletal pain care is required (68). There is an urgent need for governments, insurers, and health services to support and invest in high-value pain care, while concurrently disinvesting in low-value or no-value pain care (62). A change in the funding criterion and regulations within health systems for multidisciplinary services that aligns with and supports the use of a biopsychosocial approach will facilitate a change in the environmental context in which biopsychosocial pain care can be optimized. Hence, we incorporated the BCTs “3.1 Social support (unspecified)” and “3.2 Social support (practical)” into the synthesized framework because high value, biopsychosocial musculoskeletal pain care is the result of relationships, collaboration, coordination and authentic communication across the whole-of-health.

The availability of courses, and the re-design of curricula and capabilities/competencies across health disciplines is required to mobilise the knowledge and interpersonal skills required to support quality person-centred biopsychosocial musculoskeletal pain care. The design of value-add clinical systems learning roles as entrustable professional activities can enable healthcare students to learn tacit and contextualized knowledge. This could help bridge the gap between fulfilling a checklist of competencies and applying the knowledge and skills in dynamic, complex real-life situations (70). Here, the BCT “12.1 Restructuring the physical (learning) environment” is suggested.

Finally, the work spaces in which healthcare professionals practise is important. To implement behaviour change, introducing an environmental stimulus such as allocating a designated waiting room (with soundproof walls and a door), allows for a safe space for screening of psychosocial factors and can facilitate sensitive disclosure about patients' pain experience (64). The same contextual cues may help strengthen the context-behaviour association (71, 72) of the healthcare professional practising using a biopsychosocial approach in a safe space. Here, the BCT “7.1 Prompts/Cues” is suggested.

A worked example of how the derived framework of BCTs could be operationalised to improve adoption of biopsychosocial musculoskeletal pain care across the whole-of-health can be found in Supplementary Table S7. Supplementary Table S7 has specific examples on how to use our identified BCTs to target on healthcare professionals, educators, guideline developers, workplace managers and policymaker, in order to facilitate the implementation of biopsychosocial pain care.

### Potential strategies to empower healthcare professionals to *assess*, *identify* and *analyse* biopsychosocial factors

Though not an explicit aim of the study to answer *what* “knowledge” and “cognitive and interpersonal skills” are needed, and *how* to empower healthcare professionals to assess, identify and analyse biopsychosocial factors, the team was able to map potential strategies from best-level evidence during the inductive analysis process to derive the framework of BCTs. These suggestions are by no means comprehensive in scope but may serve as useful insights to implementation interventionists.

Review-level evidence highlights that a strong therapeutic alliance underpinned by trust, rapport, an affective bond demonstrating emotional sensitivity to patients; patient-centred empathic communication; and agreement on tasks and treatment goals can affect pain outcomes and physical functioning (78–80). Specific to patient-centred communication, strategies such as asking open-ended questions, discussing options, encouraging questions, expressing empathy and providing reassurance, explaining and providing information (59, 79), and validating the disclosure of patients (81, 82) are all important. This means biopsychosocial musculoskeletal pain care involves establishing meaningful connections with patients, shared-decision making, and supportive self-management (64, 83, 84). This will require communication behaviours synonymous to health coaching and/or motivational interviewing to navigate and optimise the clinical consultation (64, 84).

The communication behaviour in health coaching closely aligns with a recently developed classification of motivation and behaviour change techniques (MBCTs) derived from self-determination theory (73). Of note, self-determination theory is not part of the 19 theories used to formulate the BCW (23, 24) and MBCTs belong to a different taxonomy (not part of BCTTv1) (73). MBCTs offer unique insights into the specific behaviour change techniques that respond to human primacy needs of autonomy, competence and relatedness (73). Especially in persistent pain or centrally maintained pain states, there are more than biological factors driving a human pain experience (85). Restoring health and well-being requires healthcare professionals to consider these needs. Using MBCTs as a tool, or as “instructions on how to perform the communication behaviour” may support and enable healthcare professionals to better *assess* biopsychosocial factors. Behavioural counselling skills can help enable persons with chronic musculoskeletal pain to make positive lifestyle changes and encourage adherence to self-management (77). Here, the use of MBCTs may help motivate health behaviour change in patients with musculoskeletal pain. See Supplementary Table S8 for list of MBCTs.

To empower healthcare professionals to learn to *identify* and *analyse* biopsychosocial factors, a focus of intervention might consider designing educational training programs. Here the aim would be to illustrate the multidimensional interacting biopsychosocial factors associated with musculoskeletal pain and identify how, for each person, these interacting factors create a unique multidimensional experience of pain. Our previous qualitative evidence synthesis highlighted that healthcare professionals, while aware of the importance of psychosocial factors, were unclear about what those specific factors were (15). While addressing biological factors remains important, a broader view that captures the impact of psychological and social dimensions, reflects the multidimensionality of each individual's unique pain experience. During the inductive analysis process, we developed a list of recommendations to address healthcare professionals' training (see Box 1 below). This list can be further strategized, contextualized and incorporated into training curricula to enhance healthcare professionals' understanding of the common psychosocial factors associated with musculoskeletal pain presentations.

**Box 1:** A suggested list of evidence-informed strategies^*^ to help promote and enhance healthcare professionals’ awareness of psychosocial factors associated with musculoskeletal pain.

**Suggestion 1:** Applying the International Classification of Functioning, Disability and Health (ICF) framework to gauge the level of health or disability for a person’s pain presentation, by taking into account the person’s bodily function, activity limitation and participation restriction and contextual factors that might influence function (86, 87). This may increase awareness of the impact of pain on a person’s life.
**Suggestion 2:** Providing information about the social determinants of health (SDH) that can influence recovery of patients with musculoskeletal impairments (88), may increase awareness and early recognition of the contribution of SDH to disparities in musculoskeletal pain conditions, such as low back pain outcomes (89).
**Suggestion 3:** Providing evidence that early life stress, adverse childhood experiences, stressful life events, perceived injustice, and iatrogenic factors are associated with musculoskeletal pain and increased risk of developing chronic pain (90–99). Pain can be triggered by all these factors, and these factors can also lead to /prolong pain.
**Suggestion 4:** Applying a lifespan perspective to the teaching and understanding of acute, recurrent, and chronic musculoskeletal pain to raise awareness that pain can emerge, resolve, recur, and persist from childhood to old age (100, 101).
**Suggestion 5:** Incorporating medical humanities into the teaching of pain science in musculoskeletal pain may provide a more authentic and compelling understanding of peoples’ pain narratives, and a more vivid description of the impact of pain on quality of life (102, 103).
**Suggestion 6:** Providing evidence that psychological factors such as fear avoidance beliefs, increased fear of pain and pain-related anxiety are associated with greater pain intensity and disability (104–106), giving agency to enquire about these factors when assessing and managing people experiencing pain.
**Suggestion 7:** Providing evidence that chronic musculoskeletal pain is associated with higher prevalence and levels of depression, disability, decreased participation in social aspects of daily life, lower quality of life and close relationships conflicts (107), giving agency on what to expect when managing people experiencing chronic pain.

ICF, international classification of functioning, disability and health; SDH, social determinants of health.

^*^ References to inform and support suggestions are drawn from systematic reviews or best-level evidence where possible.

Practicing biopsychosocial pain care requires healthcare professionals to believe their patients about their report of pain, i.e., validation is critical. From a person-centred frame, this involves doing what is right for each person (aligned to their priorities and goals) at the right time, and taking into account relevant biological factors, their psychological wellbeing and social and environmental circumstances. It is important to educate healthcare students' and health professionals to be listen carefully to each person's pain narrative/story and work in partnership to address various contextual life events within a person-centred evidence-based framework. This approach flips the lens towards the person rather than their condition. Such an inversion that is required of the healthcare professional is not easy but can be trained (64). Here, the focus becomes training healthcare professionals to empathise with their patients, to create more authentic communication and emotional connection that builds therapeutic alliance and supports recovery.

### The biopsychosocial model of pain 40 years on: How this work improves what may be limiting implementation

Most research focus on improving the adoption of the biopsychosocial model at the micro-level, i.e., the clinical interface (11, 83, 84, 108). In this regard, we highlighted and proposed how the training of communication strategies and empathic listening (64, 81, 82) and insights from behavioural change techniques (31, 73) can help to enhance training efforts to support implementation and improve the quality of musculoskeletal pain care. At the meso- and macro-level, contextual factors and the interdependencies between various stakeholder groups in the whole-of-health within modern healthcare systems have not been adequately addressed and have not been addressed well in health systems strengthening strategies (68, 109). This may be one key to limiting effective implementation. Our work gives a refreshing whole-of-health perspective to a more-than-four-decade old biopsychosocial model of pain care.

### Implications for research and practice

Further research and road testing is required to check the validity, credibility and transferability of our derived BCT framework, including through relevant stakeholder engagement or an interdisciplinary partnership model. In this context, the evaluation of contemporary musculoskeletal models of care and policy-into-practice initiatives that incorporate a biopsychosocial approach, will be useful (109–112).

## Conclusion

From a behavioural perspective, implementation of a biopsychosocial approach to musculoskeletal pain care is a highly complex task. We have derived a conceptual model and a framework of BCTs to support future implementation efforts. Other than healthcare professionals, this requires a system-wide initiative from multi-stakeholders such as educators, to workplace managers and non-medical professions involved in healthcare (e.g., insurance workers, vocational rehabilitation providers), to guideline developers and policymakers. At the micro-level, prioritizing intervention efforts aimed at educational upskilling in a biopsychosocial approach, critical clinical reasoning and effective communication behaviours to strengthen therapeutic alliance are proposed. At the meso- and macro-level, encouraging multi-sectoral partnerships across the whole-of-health, increasing the availability of health workforce pain training programs and the re-design of curricula to strengthen interdisciplinary pain competency are crucial.

## Data Availability

The original contributions presented in the study are included in the article/supplementary material, further inquiries can be directed to the corresponding author/s. The data analyzed in this study was obtained from https://journals.lww.com/pain/Abstract/2021/08000/Barriers_and_enablers_influencing_healthcare.2.aspx, the following licenses/restrictions apply [Copyright © 2021 International Association for the Study of Pain]. Requests to access these datasets should be directed to PAIN or Wendy Ng, w.ng21@postgrad.curtin.edu.au.
